# Supplementary material for: The clinical effectiveness of one-dose vaccination with an HPV vaccine: A meta-analysis of 902,368 vaccinated women
Source: PLoS One. 2024 Jan 5;19(1):e0290808. doi: 10.1371/journal.pone.0290808 (PMC10769028; doi:10.1371/journal.pone.0290808)
Supplement: S1 Data — (DOCX) [file pone.0290808.s002.docx]

SEARCH RESULT

SYSTEMATIC REVIEW AND META ANALYSIS

Search Date : 18 November 2022

P :

I :

C :

O :

Keyterms (Best Match)

|  | **Keywords** | **Search term** | **Results** |
| --- | --- | --- | --- |
|  | HPV Vaccine | MeSH  "Papillomavirus Vaccines"[Mesh] | 9799/1848 |
|  |  | Tw  "Papillomavirus Vaccines"[tw] OR  “Vaccines, Papillomavirus”[tw] OR  “Human Papillomavirus Vaccines”[tw] OR  “Papillomavirus Vaccines, Human”[tw] OR  “Vaccines, Human Papillomavirus”[tw] OR  “Human Papilloma Virus Vaccines”[tw] OR  “HPV Vaccines”[tw] | 10571/2074 |
|  |  | Combined  "Papillomavirus Vaccines"[Mesh] OR  "Papillomavirus Vaccines"[tw] OR  “Vaccines, Papillomavirus”[tw] OR  “Human Papillomavirus Vaccines”[tw] OR  “Papillomavirus Vaccines, Human”[tw] OR  “Vaccines, Human Papillomavirus”[tw] OR  “Human Papilloma Virus Vaccines”[tw] OR  “HPV Vaccines”[tw] | 10697/2096 |
|  |  | MeSH |  |
|  |  | Tw  “One dose”[tw] OR  “Single dose”[tw] OR  “first dose”[tw] OR  "initial dose" [tw] | 90869/13383 |
|  |  | Combined |  |
| I |  | MeSH |  |
|  |  | Tw |  |
|  |  | Combined |  |
| C |  | MeSH |  |
|  |  | Tw |  |
|  |  | Combined |  |
| O |  | MeSH |  |
|  |  | Tw |  |
|  |  | Combined |  |
| PubMed Combined | | | |
| PICO Combined  ("Papillomavirus Vaccines"[Mesh] OR "Papillomavirus Vaccines"[tw] OR “Vaccines, Papillomavirus”[tw] OR “Human Papillomavirus Vaccines”[tw] OR “Papillomavirus Vaccines, Human”[tw] OR “Vaccines, Human Papillomavirus”[tw] OR “Human Papilloma Virus Vaccines”[tw] OR “HPV Vaccines”[tw]) AND (“One dose”[tw] OR “Single dose”[tw] OR “first dose”[tw] OR "initial dose" [tw]) | | | 405/115 |
| Embase Database Combined | | | |
| PICO Combined  ('Papillomavirus Vaccines'/exp OR 'Papillomavirus Vaccines' OR 'Vaccines, Papillomavirus'/exp OR 'Vaccines, Papillomavirus' OR 'Human Papillomavirus Vaccines'/exp OR 'Human Papillomavirus Vaccines' OR 'Papillomavirus Vaccines, Human'/exp OR 'Papillomavirus Vaccines, Human' OR 'Vaccines, Human Papillomavirus'/exp OR 'Vaccines, Human Papillomavirus' OR 'Human Papilloma Virus Vaccines'/exp OR 'Human Papilloma Virus Vaccines' OR 'HPV Vaccines'/exp OR 'HPV Vaccines') AND ('One dose'/exp OR 'One dose' OR 'Single dose'/exp OR 'Single Dose') | | | 494/135 |
| Combined for two databases | | |  |

Inclusion criteria

Exclusion Criteria

Total articles from two databases : 250

Number of duplicates : 74

After check duplicate : 176

Title and abstract (includen for fulltext screening) : 22

Screening fulltext:

wrong publication stype 5

Wrong study design (only protocol) 3

Wrong outcome 3

Wrong drug 1
